# COUP-TFI modifies CXCL12 and CXCR4 expression by activating EGF signaling and stimulates breast cancer cell migration

**DOI:** 10.1186/1471-2407-14-407

**Published:** 2014-06-06

**Authors:** Antoine Boudot, Gwenneg Kerdivel, Sylvain Lecomte, Gilles Flouriot, Mireille Desille, Florence Godey, Jean Leveque, Patrick Tas, Yves Le Dréan, Farzad Pakdel

**Affiliations:** 1 Institut de Recherche en Santé-Environnement-Travail (IRSET), INSERM U1085, Université de Rennes 1, Equipe TREC, Biosit, Rennes, France; 2 CHU Rennes, CRLCC Eugène Marquis et Centre de Ressources Biologiques-Santé, F-35033 Rennes, France; 3 Inserm, UMR991, Foie, Métabolismes et Cancer, F-35033 Rennes, France; 4 INSERM U1085, IRSET, University of Rennes 1, Beaulieu Campus, 35042 Rennes cedex, France; 5 Present address: Tufts Medical Center, Tufts University School of Medicine, Boston, MA, USA

**Keywords:** COUP-TF, CXCL12 signaling, Estrogen receptor, Cell migration, Breast cancer

## Abstract

**Background:**

The orphan receptors COUP-TF (chicken ovalbumin upstream promoter transcription factor) I and II are members of the nuclear receptor superfamily that play distinct and critical roles in vertebrate organogenesis. The involvement of COUP-TFs in cancer development has recently been suggested by several studies but remains poorly understood.

**Methods:**

MCF-7 breast cancer cells overexpressing COUP-TFI and human breast tumors were used to investigate the role of COUP-TFI in the regulation of CXCL12/CXCR4 signaling axis in relation to cell growth and migration. We used Immunofluorescence, western-blot, RT-PCR, Formaldehyde-assisted Isolation of Regulatory Elements (FAIRE) assays, as well as cell proliferation and migration assays.

**Results:**

Previously, we showed that COUP-TFI expression is enhanced in breast cancer compared to normal tissue. Here, we report that the CXCL12/CXCR4 signaling pathway, a crucial pathway in cell growth and migration, is an endogenous target of COUP-TFI in breast cancer cells. The overexpression of COUP-TFI in MCF-7 cells inhibits the expression of the chemokine CXCL12 and markedly enhances the expression of its receptor, CXCR4. Our results demonstrate that the modification of CXCL12/CXCR4 expression by COUP-TFI is mediated by the activation of epithelial growth factor (EGF) and the EGF receptor. Furthermore, we provide evidence that these effects of COUP-TFI increase the growth and motility of MCF-7 cells in response to CXCL12. Cell migration toward a CXCL12 gradient was inhibited by AMD3100, a specific antagonist of CXCR4, or in the presence of excess CXCL12 in the cell culture medium. The expression profiles of CXCR4, CXCR7, CXCL12, and COUP-TFI mRNA in 82 breast tumors and control non-tumor samples were measured using real-time PCR. CXCR4 expression was found to be significantly increased in the tumors and correlated with the tumor grade, whereas the expression of CXCL12 was significantly decreased in the tumors compared with the healthy samples. Significantly higher COUP-TFI mRNA expression was also detected in grade 1 tumors.

**Conclusions:**

Together, our mechanistic *in vitro* assays and in vivo results suggest that a reduction in chemokine CXCL12 expression, with an enhancement of CXCR4 expression, provoked by COUP-TFI, could be associated with an increase in the invasive potential of breast cancer cells.

## Background

During cancer progression, cancer cells first proliferate in the primary cancer site before the acquisition of the migratory behavior that leads to their spread in the body and ultimately to the development of metastasis. Estradiol (E2) and estrogen receptor alpha (ERα) play pivotal roles during ERα-positive breast cancer progression: E2-ERα signaling contributes to cell growth but prevents metastatic potential by preserving the differentiated status of the cells [1-4]. Although the loss of estrogenic signaling is generally associated with disease aggravation, the process remains poorly understood [4-7]. Indeed, many mechanisms may be involved because growth factors assume the control of cell growth and migratory capacities [1,4]. We have previously identified COUP-TFI (chicken ovalbumin upstream promoter transcription factor I) as a promoter of estrogen-independent ERα transcriptional activity in breast cancer cell lines [8,9]. Moreover, COUP-TFI was found to be overexpressed in breast tumors and to enhance the proliferation of ER-positive breast cancer cells [9]. COUP-TFI and COUP-TFII are orphan nuclear receptors that can also act by modulating other nuclear receptors, including ERα, functioning selectively as a co-activator or a co-repressor [10] to control biological processes linked to cellular growth, migration, or angiogenesis and potentially contributing to cancer progression [10-12]. Particularly, COUP-TFI expression is associated with the migration behavior of various cells during embryonic development. Accordingly, evidence from several studies supports that COUP-TFI and COUP-TFII expression in cancer cells may be associated with a dedifferentiation phenotype, the reactivation of embryonic pathways, and migration behavior, supporting the induction of aggressive characteristics in cancers [11-14]. Although COUP-TFI is suggested to be a potent mediator of cancer progression, little is known about the endogenous targets of this orphan nuclear receptor in breast cancer cells.

The chemokine CXCL12 signaling axis may represent one such axis. This signaling pathway, which is composed of the chemokine CXCL12 (also called SDF-1 for stromal cell-derived factor 1) and its receptors CXCR4 and CXCR7, play pivotal roles in the cell migration, angiogenesis, proliferation, and survival of many cancer cells, including breast cancer [15,16]. CXCR4 is typically highly expressed in metastatic cells and supports the privileged homing of these metastatic cells to specific sites where the local secretion of CXCL12 is important, namely the bone, liver, brain, and lung [17-19]. Indeed, reduction or the loss of the local secretion of CXCL12 at the tumor site can induce the emergence of metastatic cells that may spread in the organism toward endocrine sources of CXCL12 [20-22]. Although the pivotal role of the CXCL12/CXCR4 axis in cell motility and consequently in cancer metastasis in several tissues is well established, the contribution of CXCL12 *via* its receptor CXCR7 is less understood.

CXCL12 signaling may be connected to the phenotypic characteristics modified by COUP-TFI; thus, we hypothesized that COUP-TFI could target this signaling pathway in breast cancer cells. Furthermore, as the entire CXCL12/CXCR4 signaling axis is an endogenous target of E2 and is pivotal to hormonal-induced MCF-7 cell growth [23], COUP-TFI could achieve the loss of its estrogenic regulation. In the present study, we developed MCF-7 breast cancer cells overexpressing COUP-TFI protein and examined the regulation of CXCL12 signaling axis. We provide evidence that COUP-TFI increases the motility of MCF-7 ERα-positive breast cancer cells by acting on CXCL12/CXCR4 signaling as an endogenous target. The modification of CXCL12/CXCR4 expression by COUP-TFI is mediated by the activation of epithelial growth factor (EGF) and its receptor (EGFR) in MCF-7 cells. These results correlate with the expression profiles of COUP-TFI, CXCL12, and CXCR4 in breast tumors compared to healthy samples.

## Methods

### Antibodies and reagents

A goat polyclonal antibody against human CXCL12 (R&D Systems AF-310-NA), rabbit polyclonal antibody against CXCR4 (Abcam Inc. ab2074), mouse monoclonal antibody against human CXCR7/RDC1 (R&D Systems clone 11G8; MAB42273), a rabbit polyclonal antibody against COUP-TFI (Abcam Inc. ab11954) and a rabbit polyclonal antibody against HA epitope (Santa Cruz sc-805) were used for the immunofluorescence and western blot assays. A mouse polyclonal antibody against phosphorylated ERK (Santa Cruz sc-7983) and rabbit polyclonal antibody against total ERK (Santa Cruz sc-94) were used for the western blot assays.

The reagents used for treatments (17-β-estradiol (E2), ICI_182,780_ (ICI), and AMD3100) were purchased from Sigma-Aldrich Co. The recombinant CXCL12 used for the proliferation and migration assays was purchased from R&D Systems (350-NS-050).

### Cell culture and treatments

MCF-7 cells were routinely maintained in DMEM (Invitrogen) supplemented with 10% fetal bovine serum (FBS; Biowest) and antibiotics (Invitrogen) at 37°C in 5% CO_2_. Stably transfected MCF-7 clones were obtained as previously described [9]. A pool of two independent control clones and two independent COUP-TFI-overexpressing (COUP) clones were used for this study.

When treatments with steroids were required, the cells were maintained for 24 h in DMEM without phenol red (Invitrogen) supplemented with 2.5% dextran-treated charcoal-stripped FBS (dsFBS) prior to the experiments. The treatments were then performed in phenol red-free DMEM with 2.5% dsFBS and E2 (10^−8^ M), ICI (10^−6^ M), or both together for 48 hours; 0.1% ethanol was used as a control (EtOH).

### Immunofluorescence

Cells were plated on 10 mm‒diameter cover slides in 24‒well plates (5 × 10^4^ cells per well). After 48 h, the cells were fixed for 10 min in phosphate‒buffered saline (PBS) containing 4% paraformaldehyde. The cells were then permeabilized in PBS containing 0.3% Triton X‒100 for 10 min. The primary antibodies were diluted (1:100) in PBS containing 3% FCS and added to the permeabilized cells, which were incubated over night at 4°C. Dye-conjugated secondary antibodies (1:1000, Alexa Fluor, Invitrogen) were incubated 1 h at room temperature. After mounting in Vectashield® mounting medium with DAPI (Vector), images were obtained using an Imager.Z1 ApoTome AxioCam (Zeiss) epifluorescence microscope and processed with Axio Vision Software.

### RT-PCR assays

2.5 × 10^5^ cells were cultured in 6-well plates and treated as specified. Total RNA was extracted, at least in triplicate, using the Trizol™ reagent (Invitrogen) according to the manufacturer’s instructions. cDNA was generated by MMLV Reverse transcriptase (Invitrogen) using random hexamers (Promega). Quantitative real-time RT-PCR was performed using the iQ SybrGreen supermix (Bio-Rad, Hercules) and a Bio-Rad MyiQ apparatus. The primers (Proligo Primers and Probes, Boulder, CO, USA) used for the cDNA amplifications in the quantitative RT-PCR experiments are described in Table 1. GAPDH and RNA 18S were used as housekeeping genes to normalize the expression levels of the genes of interest. GAPDH was found to be appropriate for normalisation in cell lines because its expression was not affected by treatments and remained stable in control and COUP clones. For tissues, we first verified the choice of the reference gene as an internal control and its suitability in our study. Four housekeeping genes were tested (GAPDH, HPRT1, TBP and 18S RNA). The stability of these genes across different tissues and tumor grades was assessed using geNorm algorithm [24]. This software has listed HPRT1 as the best gene but HPRT1 is expressed at very low level in normal tissues and tumors, making it quite difficult to accurately quantify and not enough useful as an internal reference in our study. The second best gene, established by the software in the list, was the 18S RNA. This RNA is expressed similarly at relatively high levels in all tumors and made ideal positive control for our study. Thus, we have chosen 18S for normalization.

**Table 1:** Sequences of primers used in this study

**Gene name and symbol**	**Forward primer**	**Reverse primer**
Chemokine (C-X-C motif) receptor 4 (CXCR4)	GCCTTATCCTGCCTGGTATTGTC	GCGAAGAAAGCCAGGATGAGGAT
Chemokine (C-X-C motif) receptor 7 (CXCR7)	ACAGGCTATGACACGCACTG	ACGAGACTGACCACCCAGAC
Chemokine (C-X-C motif) ligand 12 (CXCL12)	CACCATTGAGAGGTCGGAAG	AATGAGACCCGTCTTTGCAG
Nuclear receptor subfamily 2, group F, member 1 (NR2F1) (COUP-TFI)	TACGTGAGGAGCCAGTACCC	CGATGGGGGTTTTACCTACC
Epidermal Growth Factor (EGF)	CAGGTAATGGAGCGAAGCTTTCA	GTGCATCGACATAGTTCATTCTTCTTG
Epidermal Growth Factor receptor (EGFR)	GGAGAACTGCCAGAAACTGACC	GCCTGCAGCACACTGGTTG
GlycerAldehyde-3-Phosphate DesHydrogenase (GAPDH)	GGGCATCCTGGGCTACACTG	GAGGTCCACCACCCTGTTGC
18S RNA	GCAATTATTCCCCATGAACG	AGGGCCTCACTAAACCATCC

Melting curves and PCR efficiency analyses were performed to confirm correct amplification. Each experiment was performed at least three times. Results were expressed according to the comparative Ct method (ddCt) for relative quantification of gene expression. For each sample, the difference (dCt) was calculated between Ct values obtained for target and reference amplicons. Comparative ddCt was then determined using as a reference the dCT calculated for the vehicle control sample (ethanol), and absolute values for comparative expression level were determined as equal to 2^-ddCt^.

### Protein extraction/Western blotting

Total proteins were extracted in RIPA buffer (1% NP40, 0.5% Na-deoxycholate, and 1% SDS in PBS) with an anti-protease mixture (Complete EDTA free Antiproteases, Roche) and quantified using the Bio Rad DC protein assay kit. The proteins were diluted in Laemmli buffer and denatured at 95°C; 30 μg of denatured proteins were separated on SDS polyacrylamide gels (10 and 15%), transferred to polyvinylidene difluoride membranes (Millipore), and probed with specific antibodies. The antibodies used for the Western blot assays were diluted 1:2000 (for the detection of COUP-TFI, HA, CXCL12, CXCR4 and CXCR7) or 1:5000 (for the detection of ERK or P-ERK). The detection of the immunocomplexes was performed using an enhanced chemiluminescence system (Immune Star, Bio-Rad Laboratories). For the detection of ERK activity, control and COUP cells were cultured for 48 h in phenol red-free DMEM with 0.5% dsFBS. After EGF (10^−9^ M for 5 or 10 min) or CXCL12 (200 ng/mL for 5 or 10 min) stimulation, whole-cell extracts were directly prepared in 3× Laemmli buffer. Following sonication, the protein extracts were denatured for 5 min at 95°C and analyzed as detailed above.

### Formaldehyde-assisted Isolation of Regulatory Elements (FAIRE)

FAIRE was performed as described by Eeckhoute *et al*. [25]. Briefly, asynchronously growing MCF-7 cells (60-70% confluence) treated or not for 48 h with 10^−8^ M E2 were cross-linked with 1% formaldehyde for 10 min at room temperature. Glycine was added to a final concentration of 125 mM, and the cells were rinsed with cold PBS and harvested. The cells were lysed with a solution of 1% SDS, 10 mM EDTA, and 50 mM Tris-HCl (pH 8.1) containing a protease inhibitor cocktail (Roche) and then sonicated for 14 min (30-sec on/off cycles) using a Bioruptor (Diagenode) set at the highest intensity. The soluble chromatin was subjected to three consecutive phenol-chloroform extractions (Sigma, P3803) and incubated overnight at 65°C to reverse the cross-linking. The DNA was then purified using the MinElute PCR purification kit (Qiagen). The relative enrichment of open chromatin for the *CXCL12, CXCR4* and *CXCR7* genes was quantified by real-time PCR performed using the iQ SybrGreen supermix and a Bio-Rad MyiQ apparatus. The primers used for the quantitative PCR experiments were described previously [23].

### Proliferation assay

A total of 2500 MCF-7 cells clones per well were seeded in 96-well plates and cultured in 100 μL of phenol red-free DMEM/2.5% dsFBS and EtOH or CXCL12 (200 ng/mL) for 7 days. Every 2 days, the medium was removed, and fresh treatments were performed. Proliferation was evaluated using the 3-[4,5-dimethylthiazol-2-yl]-2,5-diphenyltetrazolium bromide (MTT; Sigma) assay. 10-μL of 5 mg/mL MTT solution was added to 100 μL of culture medium in each well and incubated for 2 h at 37°C. The supernatant was removed, and the formazan formed was dissolved in 100 μL DMSO. The absorbance of each well at 570 nm was measured using a microplate reader (Bio-Rad).

### Migration assay

The cells were cultured for 48 h in phenol red-free DMEM with 5% dsFBS prior to the experiments. A total of 50,000 cells were plated in the upper chamber of a BDBiocoat control insert (BD Biosciences) in phenol red-free DMEM/0.5% dsFBS with or without AMD3100 (50 μM) or CXCL12 (200 ng/mL); phenol red-free DMEM/2.5% dsFBS with or without AMD3100 (50 μM) or CXCL12 (200 ng/mL) was added to the lower chamber. The cells were allowed to migrate for 24 h at 37°C, and the non-migrant cells were wiped off the upper chamber with a cotton swab. The insert was then placed in phenol red-free DMEM/2.5% dsFBS with calcein-AM (Invitrogen) for 1 h to stain the cells that reached the lower side of the filter. The migrant cells were then counted in 3 fields from at least 3 inserts per experimental condition.

### Ethics statement

Human samples were obtained from the processing of biological samples through the Centre de Ressources Biologiques (CRB)-Santé of Rennes (http://www.crbsante-rennes.com). We have received written informed consent from all patients for the use of their samples analyzed in this study. The research protocol was conducted under French legal guidelines and approved by the local institutional ethics committee (CPP, Comité de Protection des Personnes Ouest V de Rennes) in accordance with Helsinki Declaration. The collection of samples is reported to the Ministry of Education and Research No. DC-2008-338 which is consistent with the current ethics legislation.

### Gene expression in breast tumors

The breast tumor samples used were invasive ductal carcinoma and mostly (> 90%) ER-positive. They were divided into 20 SBR (Scarff-Bloom-Richardson grading system) Grade 1, 20 SBR Grade 2, 19 SBR Grade 3, and 23 non-tumor tissues. All samples used in this study were from fresh frozen tissues. The normal breast tissues were adjacent to the tumors but they are majority unmatched to the tumors. Total RNA was extracted using the RNeasy Mini kit (Qiagen) according to the manufacturer’s instructions, and 1 μg of total RNA was reverse transcribed with M-MLV RT (Invitrogen). Gene expression was assessed by real-time PCR (MyiQ5–Bio-Rad) with 4 ng of cDNA, 150 mM of primers (shown in Table 1), and 1× of iQ™ SYBR® Green supermix from Bio-Rad (Bio-Rad, Hercules, CA, USA). Gene expression was also measured in MCF-7 cells and served to adjust the data from different plates. The data were normalized to the expression of 18S RNA and were analyzed using IQ5 software (Bio-Rad).

### Statistical analysis

A statistical analysis was performed using Student’s t-test for most of the presented results. The values are provided as the mean ± standard error of the mean (SEM) and were considered statistically significant at *p* < 0.05. The statistical analysis for the tumor samples was performed using Minitab 16 software. The data are represented by box plots. The absence of a normal distribution of each gene for each category was verified by the Anderson-Darling normality test, and the non-parametric Mann-Whitney test was chosen to analyze our samples.

## Results

### COUP-TFI overexpression modifies the basal expression of CXCL12 and CXCR4 but not CXCR7

Our results and those of others have identified the CXCL12/CXCR4/CXCR7 axis as an important regulator of the proliferation/migration balance [17-23,26], two mechanisms that can be modulated by COUP-TFI [9,11]. For this reason, we decided to investigate the impact of COUP-TFI expression on the CXCL12 signaling axis in breast cancer cells.

**Figure 1:**
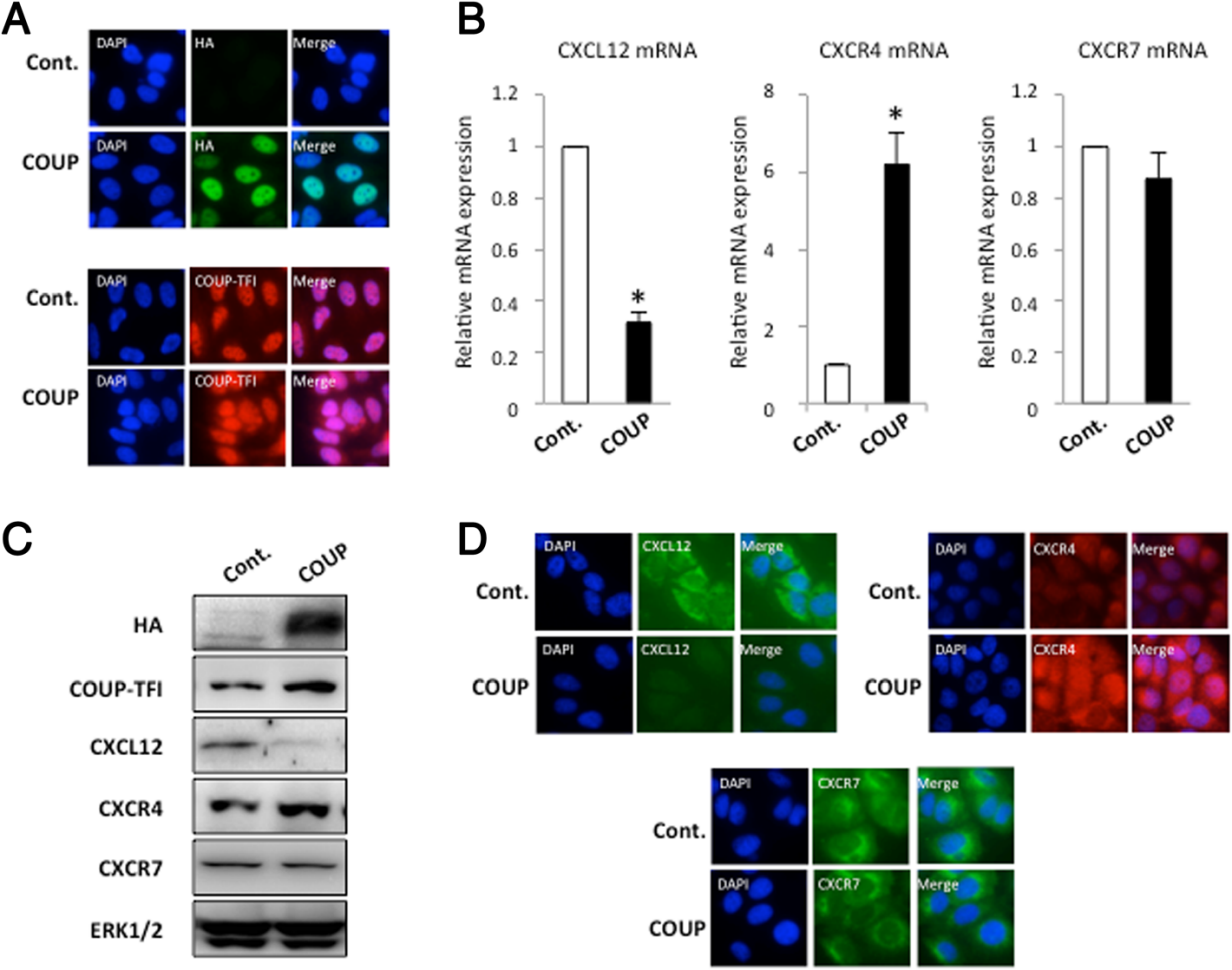
**COUP-TFI modifies the expression of the CXCL12 signaling axis in MCF-7 cells. (A)** Characterization of the control and COUP clones. An immunofluorescence cytochemistry assay was used to detect the relative expression of HA/COUP-TFI or COUP-TFI proteins in the control (Cont.) and COUP clones. The cells were fixed and processed for immunofluorescence as described in Methods; the nuclei were stained with DAPI. **(B)***CXCL12*, *CXCR4*, and *CXCR7* mRNAs were quantified by a real-time PCR analysis from two independent MCF-7 control and COUP clones. The results were normalized to *GAPDH* mRNA used as an internal control. The results were expressed as the relative mRNA expression level of *CXCL12*, *CXCR4*, or *CXCR7*. Data are the mean values ± SEM of at least three independent experiments. The asterisks indicate significant differences (*p* < 0.05) between the control and COUP clones. **(C)** The amount of intracellular HA/COUP-TFI, COUP-TFI, CXCL12, CXCR4, and CXCR7 protein was determined from whole-cell extracts of the different MCF-7 clones and compared to total ERK. A representative western blot is shown. **(D)** The control and COUP clones were fixed, and an immunofluorescence cytochemistry assay was used to detect the relative expression of CXCL12, CXCR4, and CXCR7 proteins. Staining with DAPI is also presented to visualize the nucleus of the cells.

MCF-7 cells were used as an ERα-positive breast cancer cell model: these cells weakly express COUP-TFI [9,27] and represent a good model for the study of the function of COUP-TFI upon overexpression. To investigate the influence of COUP-TFI on the regulation of *CXCL12*, *CXCR4*, and *CXCR7* gene expression, we generated MCF-7 cell clones that stably express the full-length COUP-TFI tagged with an HA epitope (named COUP); control cells were obtained from transfection with the empty vector (control). The expression of COUP-TFI was first verified in the control and COUP MCF-7 cell clones. Immunofluorescence using an antibody against the HA epitope confirmed the absence of staining in the control cells, whereas the COUP cells showed intense staining, mainly in the nucleus, corresponding to the nuclear receptor HA-COUP-TFI (Figure 1A). We also confirmed these results using an anti-COUP-TFI antibody. As shown in Figure 1, the control cells express a low level of endogenous COUP-TFI, though COUP-TFI staining is higher in the COUP cells (Figure 1A). These results were also verified by western blotting (Figure 1C). Then, the levels of CXCL12, CXCR4, and CXCR7 transcripts in the control clones and COUP clones were monitored using real-time quantitative RT-PCR. Two independent control clones and two independent COUP clones were used, and the results shown in Figure 1B represent the mean of the data. Interestingly, the overexpression of the COUP-TFI protein modified the basal expression of the CXCL12 and CXCR4 genes but did not affect CXCR7 expression (Figure 1B). Indeed, a repression of 70% of the basal expression of CXCL12 was observed in the COUP clones compared to the control clones. In contrast, we observed a 6-fold induction of the basal expression of the CXCR4 gene; CXCR7 expression was not affected when we compared COUP clones with the control clones. These results were next confirmed at the protein level using western blotting and immunofluorescence methods. The COUP clones displayed a striking reduction in CXCL12 protein expression (Figure 1C and Figure 1D), whereas the CXCR4 protein was remarkably up-regulated when compared to the control clones (Figure 1C and Figure 1D). The CXCR7 protein did not change between the different clones (Figure 1C and D). Altogether, our results suggest that COUP-TFI overexpression selectively modulates the basal expression of CXCL12/CXCR4 signaling.

**Figure 2:**
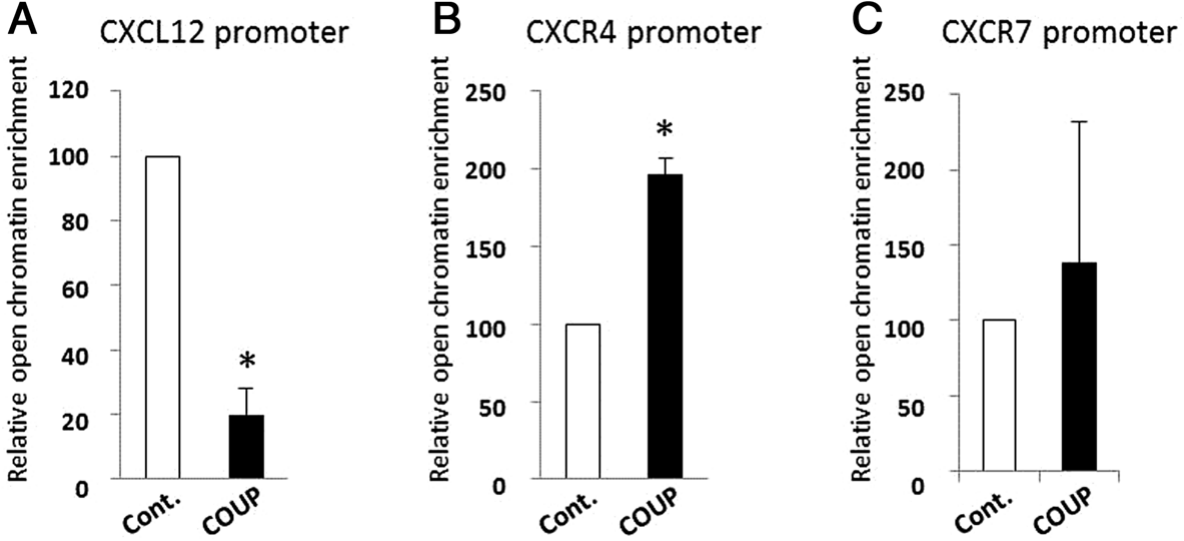
**COUP-TFI modulates the chromatin structure of the *CXCL12 *and *CXCR4 *gene promoters.** The FAIRE assay was performed as described in Methods. Real-time PCR was performed to monitor the enrichment of DNA corresponding to the proximal promoter of the *CXCL12***(A)**, the *CXCR4***(B)** and the CXCR7 **(C)** genes relative to the input chromatin from the control (Cont.) and COUP clones. The data are from triplicate samples and are representative of three separate experiments. The asterisk indicates significant differences (*p* < 0.05) between the control and COUP clones.

### Structural modifications at the *CXCL12* and *CXCR4* promoters

The level of chromatin compaction appears to be well correlated with its activity, and numerous studies have reported that active transcriptional regulatory sites are present within open chromatin regions in which the nucleosomes have been depleted .

These nucleosome-depleted genomic regions can be enriched from chromatin preparations using the FAIRE method [28]. Hence, we used FAIRE to monitor the effect of COUP-TFI on the chromatin structure of the promoters of the *CXCL12*, *CXCR4* and *CXCR7* genes in our MCF-7 clones. Interestingly, COUP-TFI overexpression led to an 80% decrease in the amount of DNA corresponding to the open *CXCL12* promoter (Figure 2A). In contrast, the *CXCR4* promoter was significantly enriched (2-fold) in the nucleosome-depleted DNA in the cells overexpressing COUP-TFI compared to the MCF-7 control cells (Figure 2B). Concerning CXCR7, no significant modifications were observed for the chromatin structure of its promoter between the control and COUP clones (Figure 2C). This result suggests that COUP-TFI selectively triggers a remodeling of the chromatin of both the *CXCL12* and *CXCR4* promoters toward a more condensed structure (*CXCL12*) or an open structure (*CXCR4*), which are well correlated to the transcriptional activities observed for these two genes.

### COUP-TFI overexpression alters CXCL12/CXCR4 estrogenic regulation

**Figure 3:**
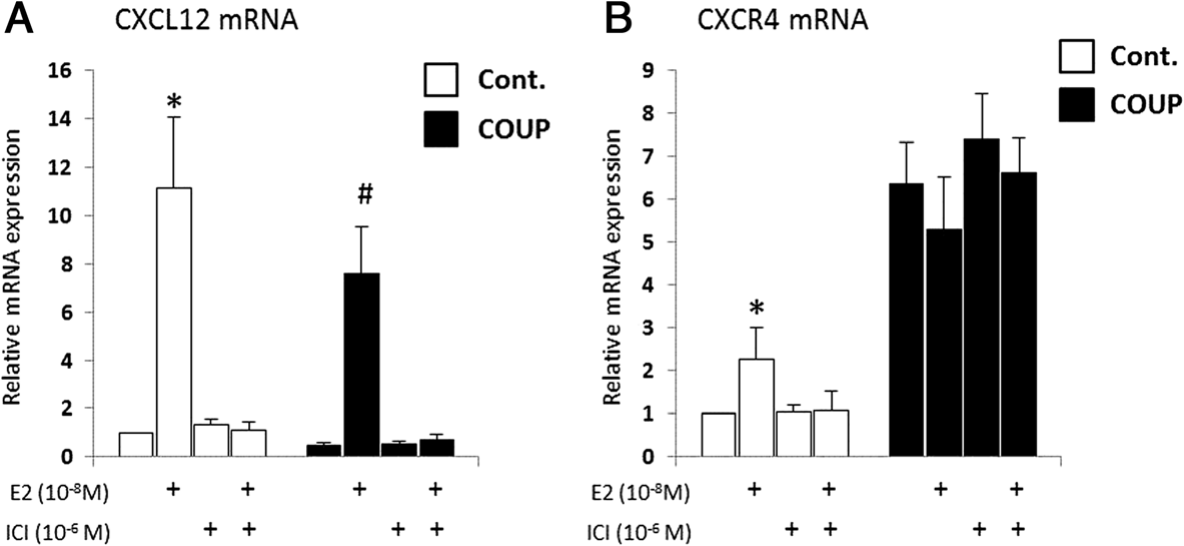
**Estrogenic regulation of CXCL12 and CXCR4 in control and COUP clones.** Control (Cont.) and COUP clones were treated with ethanol (EtOH) as the vehicle or E2 10^−8^ M and ICI 10^−6^ M alone or both together for 48 h. The *CXCL12***(A)** and *CXCR4***(B)** relative mRNA levels were monitored by a real-time PCR analysis, normalized to *GAPDH* mRNA as the internal control, and were expressed as the relative mRNA expression of *CXCL12* or *CXCR4*. Data are the mean ± SEM of at least three independent experiments. The asterisks indicate significant differences (*p* < 0.05) between the untreated and treated control clones. The pound sign indicates significant differences (*p* < 0.05) between the untreated and treated COUP clones.

Our earlier studies have shown that COUP-TFI modulates ERα transcriptional activity and is able to selectively modify the estrogenic regulation of estrogen-sensitive genes [8,9,11,29]. Because the expression of CXCL12 and CXCR4 are regulated by estrogenic signals in breast cancer cells [23], we investigated the impact of COUP-TFI on the estrogenic regulation of the CXCL12 and CXCR4 genes by treating the MCF-7 clones with 10^−8^ M E2, 10^−6^ M ICI, or both for 48 h.As expected, treatment of the control MCF-7 cells with E2 for 48 h resulted in the enhanced expression of CXCL12 (~11-fold induction, Figure 3A) and CXCR4 (~2-fold induction, Figure 3B) in comparison to the untreated and ICI-treated cells. The up-regulation of CXCL12 expression by E2 was also observed in the COUP cells treated with E2. However, the relative level of CXCL12 expression was persistently and significantly 30% lower in the COUP cells compared with the control cells (Figure 3A). CXCR4 expression was constitutively enhanced in the COUP clones, and neither E2 nor ICI, alone or in combination, had an effect on this increased CXCR4 expression (Figure 3B). This result suggests that the constitutive effect of COUP-TFI overexpression on CXCR4 mRNA is independent of ER signaling.

### COUP-TFI overexpression modulates the EGFR (ErbB-1) and MAPK signaling pathways

**Figure 4:**
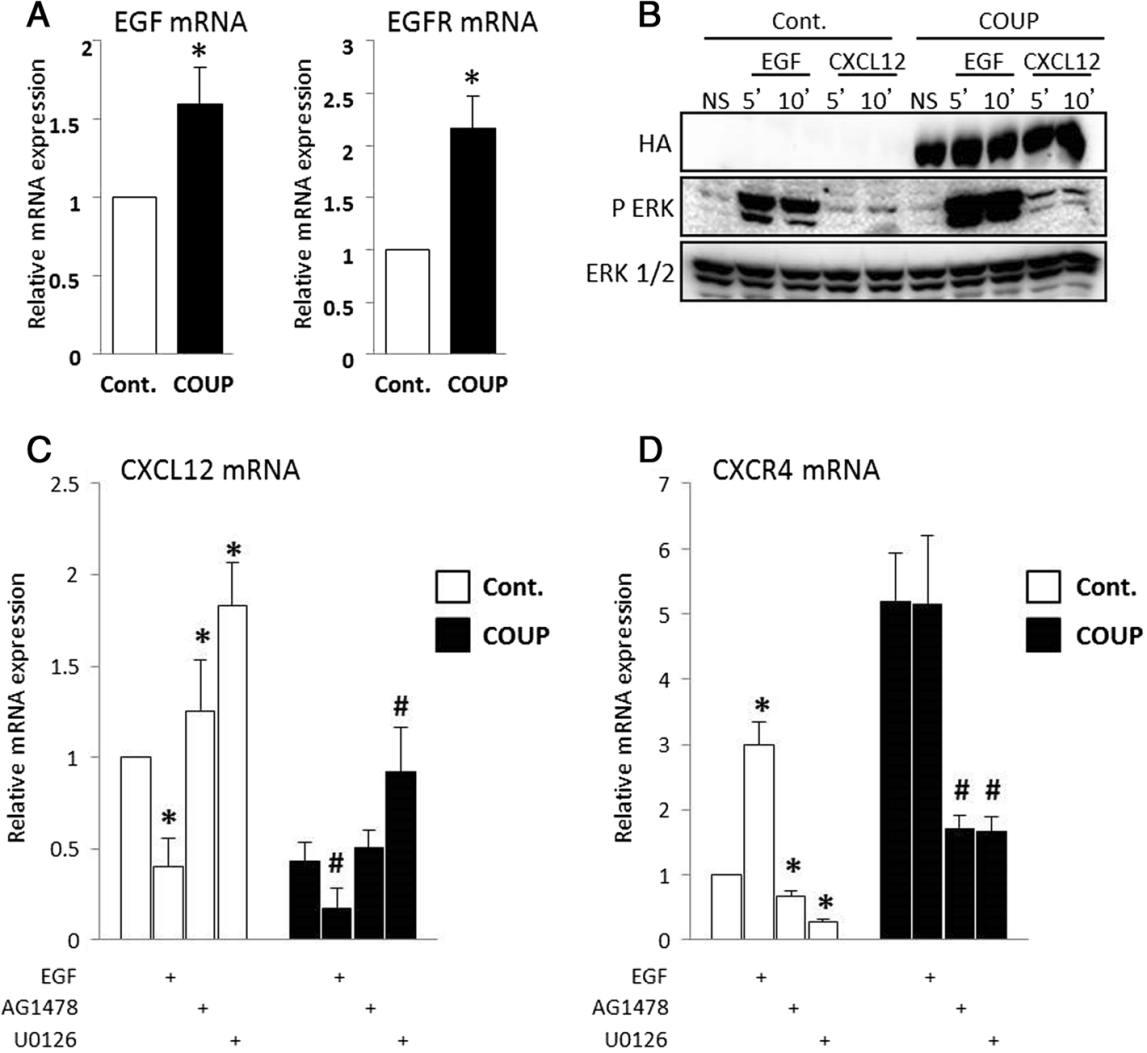
**The effect of COUP-TFI on the CXCL12/CXCR4 axis is mediated by EGF/EGFR activation. (A)** The relative expression of *EGF* and *EGFR* mRNA was monitored by a real-time PCR analysis using MCF-7 control (Cont.) and COUP clones. The results were normalized against *GAPDH* as the internal control and are expressed as the mean *EGF* or *EGFR* mRNA/GAPDH mRNA ratio ± SEM of at least three independent experiments. The asterisks indicate significant differences (*p* < 0.05) between the control and COUP clones. **(B)** ERK activation was examined in the MCF-7 control (Cont.) and COUP clones after a 5- or 10-min stimulation with EGF (10^−9^ M) or CXCL12 (200 ng/mL). Western blots were performed using antibodies against phospho-ERK (P-ERK) and total ERK (ERK1/2); a representative western blot is presented. The importance of EGFR-specific signaling and general ERK signaling on CXCL12 **(C)** and CXCR4 **(D)** regulation was assayed by treating the cells with EGF (10^−9^ M), AG1478 (25 μM), or U0126 (25 μM) for 48 h. The *CXCL12* and *CXCR4* relative mRNA levels were monitored by the real-time PCR analysis, normalized to *GAPDH* mRNA as the internal control, and were expressed as the relative mRNA expression of *CXCL12* or *CXCR4*. Data are the mean ± SEM of at least three independent experiments. The asterisks indicate significant differences (*p* < 0.05) between the untreated and treated control clones. The pound sign indicates significant differences (*p* < 0.05) between the untreated and treated COUP clones.

The growth factor control of cell fate is a pivotal step in cancer progression; indeed, the high expression of epidermal growth factor receptor (EGFR) in cancers has been associated with metastatic tumors and poor clinical outcomes. Additionally, EGFR signaling was recently linked to CXCR4 signaling [30]. Therefore, we evaluated the effect of COUP-TFI on the expression of EGF and EGFR (ErbB-1) in MCF-7 clones (Figure 4A) and found that COUP-TFI overexpression increased both EGF and EGFR expression by 1.8 to 2 times, respectively, in comparison to the MCF-7 control cells. Moreover, our earlier studies have shown that COUP-TFI was able to interplay with the MAPK pathway, enhancing ERK activity [8,9]. We confirmed this observation by analyzing ERK protein phosphorylation after the EGF stimulation of COUP cells in comparison to the control cells (Figure 4B). To further investigate whether the up-regulation of EGF signaling by COUP-TFI could be linked to the changes in *CXCL12* and *CXCR4* gene expression, treatments with EGF and selective inhibitors for EGFR (AG1478) and MEK (U0126) signaling were performed. Interestingly, EGF treatment significantly decreased CXCL12 expression in both the control and COUP clones (Figure 4C), whereas the inhibition of EGFR signaling by AG1478 and U0126 led to a slight but significant elevation in CXCL12 expression in both cells. The expression profile of CXCL12 was lower under all conditions in the MCF-7 cells overexpressing COUP-TFI. Furthermore, EGF treatment increased CXCR4 mRNA expression in the control cells but had no effect on the increased CXCR4 expression in the COUP cells (Figure 4D). Treatments with EGFR and MEK inhibitors decreased CXCR4 expression, reaching a lower level of expression than under the basal conditions. Indeed, the inhibition of EGFR and MAPK signaling abolished the stimulation effect of COUP-TFI on CXCR4 expression.

### COUP-TFI overexpression modifies cells response to CXCL12 signal

**Figure 5:**
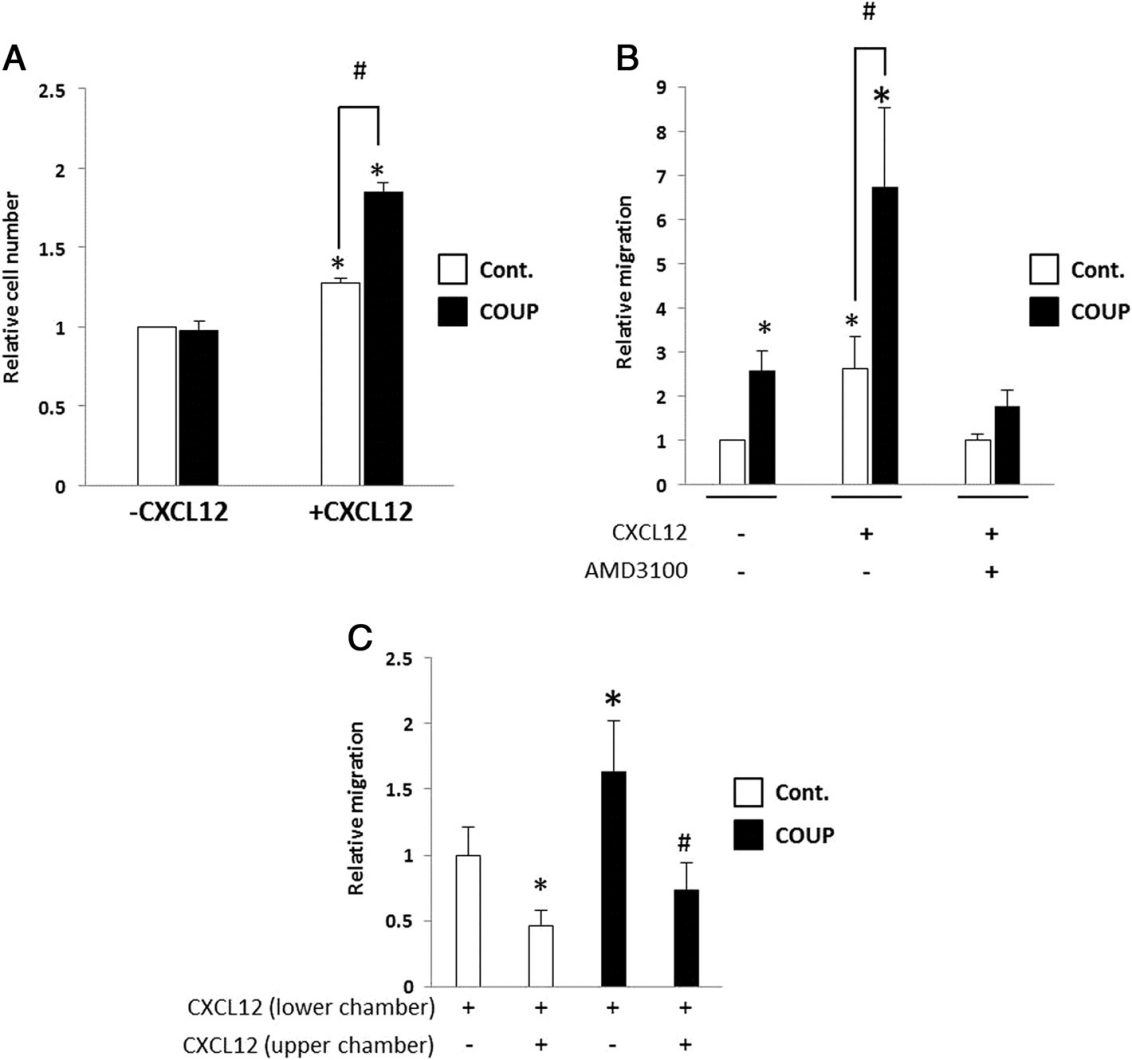
**COUP-TFI overexpression influences cellular responses to CXCL12. (A)** The relative growth of the control (Cont.) and COUP clones was assayed with or without CXCL12 treatment for 7 days. The basal and CXCL12-induced cell growth were evaluated by MTT assays (n = 6) and determined in three independent experiments. The results are expressed as the relative cell number obtained when the control cells were treated with the vehicle control. Significant differences between the unstimulated control cells and the other conditions (*p* < 0.05) are indicated with an asterisk. Significant differences between stimulated control cells and stimulated COUP clones (*p < 0.05*) are indicated with a pound sign. **(B)** The migratory capacity of control (Cont.) and COUP clones was analyzed. The cells were maintained in phenol red-free DMEM/2.5% dsFBS for 48 h and then seeded in phenol red-free DMEM/0.5% dsFBS in the upper chamber of a PET 8-μm pore insert. The cells were allowed to migrate for 24 h toward the phenol red-free DMEM/2.5% dsFBS medium complemented or not with CXCL12 (200 ng/mL) and AMD3100 (50 μM). **(C)** CXCL12 was also added to the culture medium in the upper chamber prior to migration. The results are expressed as the mean ± SEM of the relative number of migratory cells compared to the basal migration of the control cells measured in three independent experiments. The asterisks indicate significant differences (*p* < 0.05) from the basal migration of the control clones. The pound sign indicates significant differences (*p* < 0.05) between two conditions linked by black lines.

The CXCL12/CXCR4 axis plays major roles in breast cancer cell proliferation, migration, and invasion; thus, we analyzed the CXCL12-mediated growth and motility of MCF-7 cells overexpressing COUP-TFI (Figure 5). First, we tested the control and COUP cells for a proliferative response to CXCL12 treatment by exposing the cells to CXCL12 (200 ng/mL) for 7 days and quantifying the total cell number (Figure 5A). No significant differences were observed with regard to the basal growth of the control and COUP cells; however, when treated with CXCL12, both cells proliferated significantly more than under the control condition. Furthermore, the COUP cells displayed a significantly higher proliferative response to the CXCL12 treatment than the control cells.The cell migratory behavior was then assayed. We analyzed the capacity of the control and COUP cells to migrate through a PET membrane with an 8-μm filter pore toward a low serum concentration medium, which represented the “basal” migration, or toward a CXCL12 gradient, which corresponded to the “induced” migration (Figure 5B). After a 24-h period, the relative number of basal migrant cells was almost twice as high for the COUP cells than the control cells. Moreover, when CXCL12 (200 ng/mL) was added to the lower chamber, the migration of the control and COUP cells increased versus the basal condition. The relative induced migration of the COUP cells was 3 times higher than that of the control cells. Interestingly, the specific CXCR4 inhibitor AMD3100 completely abolished the CXCL12-induced migration observed in the control and COUP cells. No significant difference in migration capability between the two clones was detected.Next, we tested the hypothesis that the reduction of CXCL12 by the COUP cells is important for their migration toward a CXCL12 gradient. We therefore added human recombinant CXCL12 to both the upper chamber and the lower chamber. Under these conditions, the migratory behavior of the control and COUP cells was dramatically altered, with both clones displaying an approximately similar migration potential (Figure 5C). Thus, disruption of the CXCL12 gradient by ectopic CXCL12 added to the upper chamber prior to the migration test hampered the migration of both the control and COUP clones.

### CXCL12/CXCR4/CXCR7 and COUP-TFI mRNA expression in breast tumors

**Figure 6:**
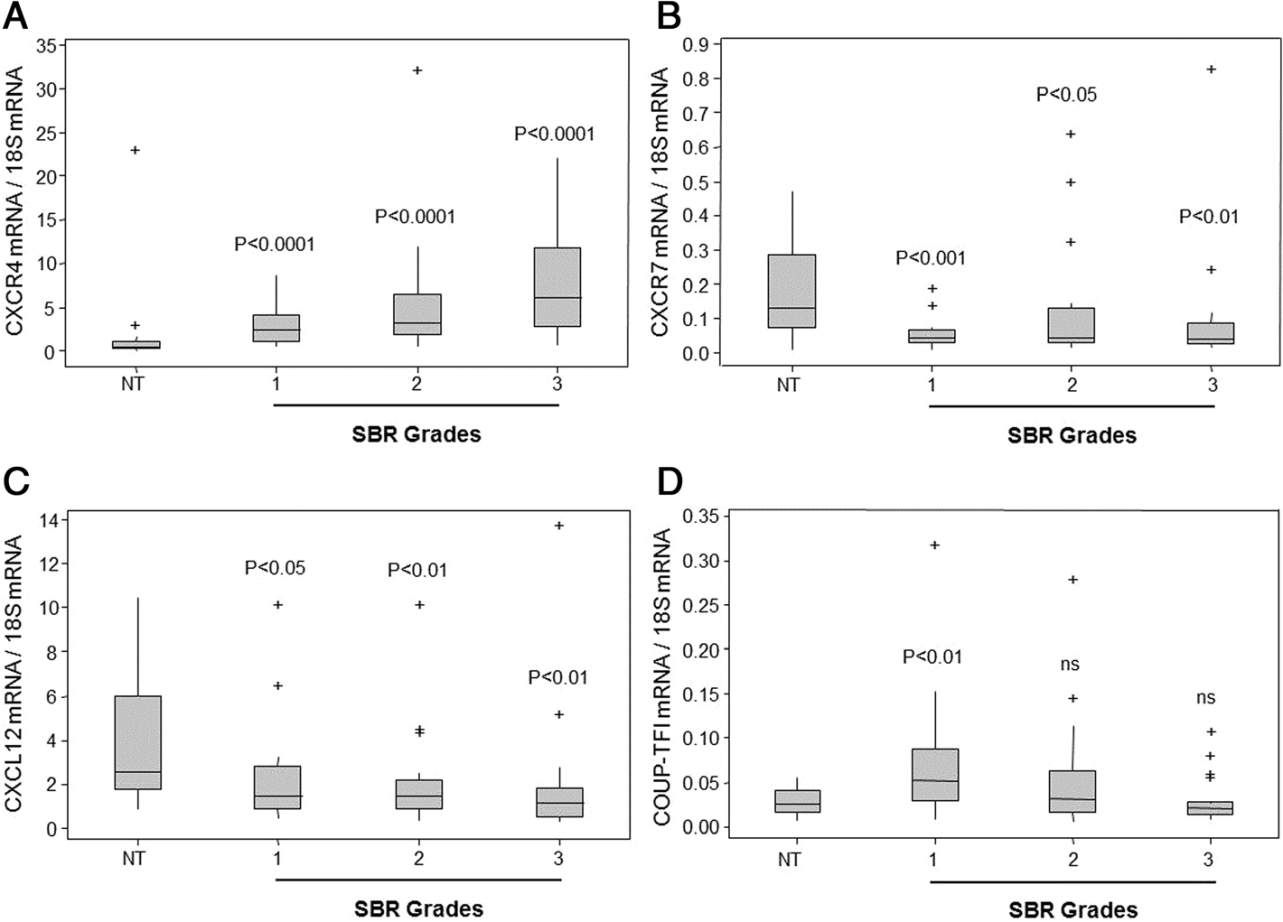
**Box plots of CXCR4, CXCR7, CXCL12, and COUP-TFI mRNA expression in breast cancer and normal tissue.** CXCR4 **(A)**, CXCR7 **(B)**, CXCL12 **(C)**, and COUP-TFI **(D)** mRNA expression was measured by real-time PCR in 23 normal breast tissue samples (NT), in 20 SBR grades 1 and 2, and in 19 SBR grades 3. The expression level was normalized by 18S RNA expression and analyzed with IQ5 software (Bio-Rad). The data are presented as whisker plots in which the horizontal bar represents the median, the grey boxes are the 25^th^ and 75^th^ percentiles, the vertical bar is the standard deviation, and the plus signs are the extreme points. All the Mann-Whitney tests were performed with Minitab 16 software, and the *p* value is indicated on the different graphs (ns denotes non-significant).

The expression profiles of CXCR4, CXCR7, CXCL12, and COUP-TFI in breast cancer cells from patients exhibiting different tumor grades (82 breast tumors and control non-tumor samples) were measured using real-time PCR (Figure 6). As depicted in Figure 6A, the level of CXCR4 mRNA was found to be significantly increased in the tumors compared to the healthy samples (p < 0.0001). A two-sided Pearson correlation was performed to seek whether a correlation exists between CXCR4 expression and the tumor grades. Indeed, we have found a strong correlation between CXCR4 expression and tumor grade (p-value = 0.000085, ρ = 0.4201 at the 95% confidence interval [0.2235; 0.5839]). Conversely, the expression of CXCR7 and CXCL12 transcripts (Figure 6B and C, respectively) was significantly decreased in the tumors compared to the healthy samples. As expected, COUP-TFI (Figure 6D) was found to be significantly overexpressed in the grade 1 tumors compared to normal tissues (p < 0.01) though was rather similar in the grade 2 and 3 tumors compared to that found in the normal samples. We have also performed a two-sided Pearson correlation analysis to determine if the relative expression of CXCR4, CXCR7 and CXCL12 in tumours is associated with the relative expression of COUP-TFI. This analysis showed a significant correlation for CXCR4/COUP-TFI (p-value = 0.029, ρ = 0.2405 at the 95% confidence interval [0.0248; 0.4348]), CXCR7/COUP-TFI (p-value = 0.0042, ρ = 0.3129 at the 95% confidence interval [0.1029 ; 0.4962]) and CXCL12/COUP-TFI (p-value = 0.030, ρ = 0.2387 at the 95% confidence interval [0.0229; 0.4333]). Moreover, our *in vitro* observations correlate well with these results, indicating that the expression of COUP-TFI and CXCR4 are enhanced, with the expression of CXCL12 declining, during cell transformation, resulting in the progression to a cancerous state.

## Discussion

The contribution of COUP-TFI to cancer progression is poorly understood. Nevertheless, this orphan nuclear receptor is known to participate in many biological processes connected to normal or pathological cell proliferation, survival, or migration (for a review, see [11]). Previous studies have established that COUP-TFs can modulate estrogen signaling, contributing to phenotypical changes in breast cancer cells [9,31,32]. Moreover, our previous study suggested that the overexpression of COUP-TFI in breast tumor cells may contribute to the loss of the epithelial phenotype and acquisition of mesenchymal characteristics [9]. In the present study, we identified CXCL12/CXCR4 signaling as an endogenous target of COUP-TFI, which could explain some of these phenotypical deviations. We demonstrated that COUP-TFI overexpression selectively and differentially alters the expression of the CXCL12 and CXCR4 genes: the basal level of CXCL12 was reduced, whereas CXCR4 basal expression was up-regulated. It was also noted that COUP-TFI disturbs the estrogenic regulation of CXCL12 and CXCR4 in MCF-7 cells, supporting the idea that COUP-TFI leads to a loss of E2 dependency in breast cancer cells. Interestingly, our data show that COUP-TFI impacts the chromatin condensation state of the proximal promoters of the *CXCL12* and *CXCR4* genes. These modifications of the chromatin structure are known to correlate with the transcriptional potential of regulatory elements and could also suggest epigenetic modifications induced by COUP-TFI. However, the precise molecular mechanisms of COUP-TFI action on the basal and E2-dependent regulation of CXCL12/CXCR4 remain to be determined.

Cancer progression is frequently associated with growth factor-induced control of cell growth and migration [33]. Notably, cross-talk between the EGFR family and E2 signaling is often associated with the loss of hormonal control of cancer cell growth and the acquisition of metastatic potential [34]. CXCR4 induction is one of the identified mechanisms for the growth factor control in cancer cells, which supports the migration of cancer cells [30]. COUP-TFI has been shown to interact with the MAPK pathway, leading to the activation of ERK activity ([8] and herein). Here, we established that COUP-TFI overexpression leads to an increase in EGF and EGFR relative expression that could, in part, explain the effect of COUP-TFI in the activation of ERK signaling activity. Our results support that the induction of MAPK activity, presumably *via* EGF signaling, is responsible for the constitutive induction effected by COUP-TFI on CXCR4 expression. Moreover, our results show for the first time that CXCL12 expression is negatively regulated by EGF signaling in breast cancer cells. We also observed similar results when the cells were treated with different serum concentrations (data not shown). Interestingly, these data are in good agreement with several studies related to stem cell homing/mobilization in bone marrow that have reported that many growth factors can down-regulate the local secretion of CXCL12, thereby promoting stem cell mobilization toward the peripheral blood [35-37]. Taken together, our results support the idea that COUP-TFI can differentially impact CXCL12 and CXCR4 basal expression by activating the ERK pathway. The constitutive activation of a transcription factor—proposed not to be ERα, given our observation that ICI treatment did not impact CXCR4 expression in COUP clones—by the MAPK pathway could explain the induction of CXCR4 expression. It was previously observed that hypoxia-inducible factor 1 alpha (HIF1-α) is induced by EGFR constitutive signaling, leading to CXCR4 up-regulation [30].

The chemokine network and CXCL12/CXCR4 signaling in particular, as well as EGFR signaling are involved in many aspects of cancer biology, including growth and metastasis [7,34,38]. Indeed, there are many evidences of the essential role of CXCR4 in the enhanced invasion of several types of cancer [39-41]. Furthermore, the down-regulation of CXCL12 expression by promoter hypermethylation has been associated with increased metastatic potential in mammary carcinoma cells [22] by the loss of autocrine and paracrine CXCL12 retention at the primary tumor site. Our results demonstrate a higher proliferative response to CXCL12 treatment by COUP clones compared to control clones, which could be due to the higher activation of ERK signaling observed after CXCL12 treatment. We also found that the COUP clones exhibited a better migration behavior than the control clones when migrating toward a serum-complemented medium or in response to a CXCL12 chemotactic gradient. Our results suggest that this higher migration behavior is due to the enhanced CXCR4 expression. In addition, we observed that ectopic CXCL12 added to the upper chamber prior to the migration test hampered the migration of both the control and COUP clones. This finding is in good agreement with a study from Zabel *et al.*, who suggested that the reduction in CXCL12-triggered migration by the additional CXCL12 within the cells could possibly be explained by the desensitization of CXCR4 or disruption of the chemokine gradient [42]. Moreover, the down-regulation of CXCL12 was previously reported to be necessary to allow the emergence of metastatic cells *in vivo*[20,21]. Taken together, our results suggest that the down-regulation of CXCL12 induced by COUP-TFI overexpression could be associated, together with the elevation in CXCR4 expression, with increased migration behavior. In other words, we propose that the opposite action of COUP-TFI on CXCL12 and CXCR4 expression enhances the migration capacity of cancer cells through an increase in sensitivity to exogenous CXCL12 and by limiting the autocrine retention effect of CXCL12. Moreover, enhanced EGFR signaling activity was reported to contribute to cancer progression from various origins through the elevation of cancer cell survival, proliferation, and migration [38]. Our results support that, by repressing CXCL12 expression and inducing CXCR4 expression, the growth factor regulation of CXCL12 signaling could trigger these effects, as was observed during stem cell mobilization from the bone marrow to peripheral blood [43].

Our previous immunohistochemistry data indicated that COUP-TFI is overexpressed in cancer compared to normal breast tissues [9]. We also showed that COUP-TFI expression increased in dedifferentitiated ER-negative breast cancer cell lines compared to differentiated ER-positive cell lines. This was correlated to protein markers of dedifferentiated phenotype, for instance E-cadherin silencing and vimentin expression [9]. A limitation of our study is that it was only performed in MCF-7 cell line. However, it is of interest to note that COUP-TFI represses in vitro the expression of type VII collagen in different human cell lines [44]. Moreover, cell contact stability was reported to be affected by COUP-TFI overexpression in fibroblast cells, most likely because of alteration of cell attachment proteins expression [45]. COUP-TFII has also been reported to be overexpressed in breast cancer epithelia [12]. COUP-TFII over expression was furthermore associated to poor clinical outcome and to invasive behavior of metastatic cells in lymph nodes [12]. However, in this study, our quantitative RT-PCRs revealed a significant augmentation of COUP-TFI mRNA expression only in grade 1 tumors, whereas grade 2 and 3 tumors exhibited expression of COUP-TFI mRNA that was similar to that observed in the normal tissues. Although, further investigation, particularly by immunohistochemistry, is necessary to reveal COUP-TFI staining in low and high grade tumor biopsies, the discrepancy between transcript and protein levels, may argue for the consequence of additional control mechanisms besides transcription. This may be attributed to differences in the mRNA and protein turn over or could originate from different translational mechanisms that may selectively stabilize COUP-TFI protein. Indeed, the expression levels of a protein depend not only on transcription rates of the gene, but also on additional control mechanisms, such as nuclear export, mRNA localization and stability, translational regulation and protein degradation [46]. Deregulation of certain of these mechanisms in cancer cells may explain this discrepancy; however, more investigations will be needed to establish that. Interestingly, our in vitro results showed that COUP-TFI overexpression does abolish E2 control of CXCR4 expression and partially reduces CXCL12 regulation. The expression profiles of CXCL12, CXCR4 and CXCR7 in breast cancer biopsies are almost identical to that obtained when we overexpressed COUP-TFI in MCF-7 cancer cells, suggesting that our in-vitro results might have a clinical relevance. It should be investigated whether increasing the expression of COUP-TFI protein during cancer progression could in fact participate in the development of hormone resistance and favor the growth and migration capacity of tumor cells. Recent studies have reported that the COUP-TFII expression level is increased in several different cancer cells, such as breast, prostate, and ovary cancers [12-14]. These studies have also shown that the overexpression of COUP-TFII is associated with a significantly shorter disease-free survival. Indeed, the overexpression of COUP-TFII in prostate cells promotes tumorigenesis and induces an aggressive metastasis characteristic in tumors by inhibiting the TGF-β-induced growth barrier [13].

## Conclusion

In summary, we identified the CXCL12 signaling axis as an endogenous target of the orphan nuclear receptor COUP-TFI. The effect of COUP-TFI is mediated by the induction of MAPK signaling and leads to enhanced growth and migration capacity in cancer cells in response to CXCL12. Although the clinical importance of these observations should be investigated further, our results predict that the disruption of COUP-TFI in breast cancer may result in the reduction of the metastatic potential of the cells.

## Abbreviations

COUP-TF: Chicken ovalbumin upstream promoter transcription factor; EGF: Epidermal growth factor; EGFR: Epidermal growth factor receptor; E2: 17-β-estradiol; ER: Estrogen receptor.

## Competing interests

The authors declare that they have no competing interests.

## Authors’ contributions

AB, GK, GF, FP performed the establishment and characterization of stable cellular clones, immunofluorescence, gene expression analysis and cell growth and migration assays. MD, performed RNA extractions from normal and cancerous breast tissues for quantitative RT-PCR analysis. SL and YLD, designed and carried out the RT-PCR amplification of CXCR4, CXCR7, CXCL12 and COUP-TFI in breast tissue samples and analyzed their expression levels. FG, JL and PT performed the characterization tumoral and non-tumoral mammary gland and defined the SBR Grade of ductal carcinoma in situ. FP designed, supervised and coordinated the study, participated in the design of all the experiments. AB and FP drafted the manuscript. All authors read and approved the final manuscript.

## Pre-publication history

The pre-publication history for this paper can be accessed here:

http://www.biomedcentral.com/1471-2407/14/407/prepub
